# Patients’ perspective on the therapeutic relationship and session quality: the central role of alliance

**DOI:** 10.3389/fpsyg.2024.1367516

**Published:** 2024-08-12

**Authors:** Alberto Stefana, Paolo Fusar-Poli, Eduard Vieta, Eric A. Youngstrom

**Affiliations:** ^1^Department of Brain and Behavioral Sciences, University of Pavia, Pavia, Italy; ^2^OASIS Service, South London and Maudsley NHS Foundation Trust, London, United Kingdom; ^3^Early Psychosis: Interventions and Clinical-detection (EPIC) Lab, Department of Psychosis Studies, Institute of Psychiatry, Psychology & Neuroscience, King’s College London, London, United Kingdom; ^4^Bipolar and Depressive Disorders Unit, Hospital Clinic, IDIBAPS, CIBERSAM, Institute of Neuroscience, University of Barcelona, Barcelona, Catalonia, Spain; ^5^Institute for Mental and Behavioral Health Research, Nationwide Children’s Hospital and Department of Psychiatry, The Ohio State University, Columbus, OH, United States; ^6^Helping Give Away Psychological Science, Chapel Hill, NC, United States

**Keywords:** working alliance, real relationship, affective reaction, therapeutic relationship, session outcome, patients’ perspective

## Abstract

In this study, we examined how four components of the therapeutic relationship—working alliance, real relationship, and positive and negative affective reactions of the patient toward their therapist—relate to each other and to the psychotherapy session outcome, from the patient’s point of view. Our simple comprised 700 adult patients in individual psychotherapy who were recruited and participated online. They underwent a baseline evaluation of their most recent therapy session, which encompassed a series of validated self-report measures focused on specific elements of the therapeutic relationship. The results revealed that, from the patient’s perspective, working alliance, real relationship, and positive affective reactions toward the therapist were positively correlated with session outcome, while negative affective reactions were negatively correlated. All components predicted session outcome when simultaneously included in a regression model. Collectively, these four components accounted for 30% of the variance in session outcome. Factor analysis revealed four distinct factors, underlying perceptions of the therapeutic relationship. Notably, the bond dimension of the alliance was sufficiently different from the task and goal dimensions, warranting consideration as a distinct construct. These findings, although cross-sectional, lay the groundwork for a more nuanced investigation of multiple dimensions of the therapeutic relationship.

## Introduction

The patient–therapist relationship is a fundamental component of psychotherapy (Høglend, 2014; Norcross and Lambert, 2018) and any intervention focused on mental health (San and Arranz, 2023). Meta–analytic estimates show that it accounts for approximately 15% of the total variance in adult psychotherapy outcomes, with the patient contributing 30%, the therapist 7%, and the specific treatment method ranging from 0 to 10% (Norcross and Lambert, 2019; see also Wampold and Imel, 2015). These data underscore the importance of investigating underexplored facets and dynamics of the therapeutic relationship (TR). Some experts advocate a transition from a holistic analysis to a more detailed exploration of its specific elements (Horvath, 2009; Bhatia and Gelso, 2018).

Two models that provide a nuanced understanding of TR are Bruce Wampold’s contextual model of psychotherapy (Wampold and Budge, 2012; Wampold and Imel, 2015) and Charles Gelso’s tripartite model of the TR (Gelso, 2014; Gelso, 2018). The contextual model, also known as the “common factors” model, posits that psychotherapy is based on an initial patient–therapist bond and unfolds through three pathways (Wampold, 2017): (a) The real relationship between the patient and the therapist provides the patient with an empathic and caring connection, beneficial to his/her health. (b) The therapist helps the patient understand the origins of their mental disorder and offers ways to cope and overcome their difficulties, fostering hope that they will be successful in completing therapy tasks and managing their problems. (c) Therapy–specific techniques and strategies can create expectations in the patient and facilitate healthy behavior changes. A psychotherapeutic treatment that incorporates these three pathways to some extent will be effective. The significance of these common factors is well-supported by empirical evidence and is crucial across various therapeutic approaches (Bailey and Ogles, 2023).

Within this metamodel of psychotherapy, a model of the TR can be embedded. The tripartite model identifies three interconnected elements: the real relationship, the working alliance, and the transference–countertransference configuration. Evidence indicates that the tripartite model predicts 27% of the variance in session outcomes, however, factor analysis revealed that items from the four therapeutic relationship measures emerged as four separate factors, albeit with some degree of overlap (Bhatia and Gelso, 2018).

Although individual studies have examined elements of the therapeutic relationship (TR) as components of the therapeutic process, few have explored the interplay between these elements, often relying on the therapist’s perspective. Consequently, patient ratings can provide valuable information to theoretically model the TR. In this context, our research represents an opportunity to enhance the understanding of the TR by integrating patient feedback. Specifically, our objective was to explore a quadripartite model (different from that theorized by Charles Gelso) of the TR from the perspective of patients, encompassing the working alliance, the real relationship, and positive and negative affective reactions toward the therapist. Theoretically, these affective reactions can be seen as a conscious phenotype of what some contemporary psychoanalysts call transference (Westen and Gabbard, 2002; Bradley et al., 2005). Specifically, our exploration focused on three broad domains:

- The relationship between patient–rated working alliance, real relationship, positive and negative affective reactions, and patient–rated session outcome.
- The interrelations among the four components of the TR.
- The grouping of items from the measures of the four components and the factors that emerged from this merged item pool through exploratory factor analysis.

## Methods

### Dataset

This research report outlines a secondary analysis of baseline data from a longitudinal study, as described in the study protocol by Stefana et al. (2024c). The Institutional Review Board of the University of North Carolina at Chapel Hill (UNC-CH) approved the study (IRB number: 23–0216; approval date: March 6, 2023).

### Participants

Participants in the study were 700 adults who underwent individual psychotherapy in the United States. Most participants (70%) received treatment in private practice settings. The remaining participants were distributed among private health institutions (11%), public health institutions (10%), and other settings (9%), such as university counseling centers. The demographic distribution included 81% females (*n* = 564), with 74% (*n* = 512) identifying with woman gender. The primary age groups represented were 23–29 years (20%, *n* = 142) and 30–39 years (28%, *n* = 193). The majority of ethnicities were Caucasian, comprising 81% (*n* = 566) of the participants. A predominant proportion, 84% (*n* = 590), had been diagnosed with at least one psychiatric ailment, with anxiety (66%, *n* = 464) and unipolar depression (56%, *n* = 391) being the most prevalent conditions. The patients received different types of psychotherapy. Table 1 details the demographic, contextual, and therapeutic characteristics of the sample. All information, including psychiatric diagnosis, was self-reported.

**Table 1:** Demographics, contextual, and treatment characteristics of participating patients (*N* = 700).

Demographics	% (*n*)
Age (years)	
18–22	9% (66)
23–29	20% (142)
30–39	28% (193)
40–49	16% (109)
50–59	14% (99)
≥ 60	13% (91)
Biological sex	
Female	81% (564)
Male	18% (128)
Intersex	0% (1)
I prefer not to say	1% (7)
Gender	
Woman	74% (512)
Man	19% (132)
Non–binary	7% (46)
I prefer not to say	1% (6)
Education	
Less than high school	0% (2)
High school graduate	3% (24)
Some college	19% (136)
2–year degree	9% (64)
4–year degree	33% (231)
Professional degree	28% (195)
Doctorate	7% (48)
Ethnicity	
White	81% (566)
Black or African American	10% (68)
Asian	4% (29)
Other	6% (37)
Clinical characteristics ^a^	
Any psychiatric disorder	84% (590)
Any anxiety disorder	66% (464)
Any (unipolar) depressive disorder	56% (391)
Any trauma– and stressor–related disorders	35% (244)
Any neurodevelopmental disorder	24% (165)
Any bipolar or related disorder	13% (88)
Any eating disorder	10% (71)
Any disruptive behavior and dissocial disorder	2% (15)
Schizophrenia or any other psychotic disorders	1% (9)
Any cluster A personality disorder	0% (3)
Any cluster B personality disorder	6% (43)
Any cluster C personality disorder	6% (41)
Treatment characteristics	
In psychotherapy from	
0 to 3 months	14% (99)
4 to 6 months	14% (96)
7 to 12 months	11% (79)
13 to 24 months	13% (94)
> 24 months	47% (332)
Session frequency	
1 or less per month	19% (130)
2 to 3 per month	39% (276)
1 per week	38% (267)
2 or more per week	4% (27)
Session attendance	
Video call	53% (369)
In person face to face	36% (251)
Telephone call	8% (59)
In person on the couch	3% (21)
Therapist biological sex (Female)	81% (565)

^a^N sums to more than 700 because cases could have more than one diagnosis.

### Measures

#### Sociodemographic and clinical domain

A *sociodemographic and clinical data* form was specifically designed for this study to capture the information reported in Table 1.

#### Therapeutic relationship domain

The *Working Alliance Inventory–Short Revised* (WAI–SR) (Hatcher and Gillaspy, 2006), a 12–item self–report measure of the working alliance in psychotherapy sessions. WAI–SR is based on Bordin (1979) theory of the working alliance and encompasses three subscales with four items each: agreement on therapy tasks, agreement on therapy goals, and the establishment of an affective bond between the patient and the therapist. The items are rated on a Likert scale ranging from 0 (“Not at all”) to 5 (“Completely”). In this study, the WAI–SR total scale showed an internal consistency Cronbach’s alpha value of 0.95. Furthermore, in previous studies, the WAI–SR has shown good convergent validity, correlated well with other established measures such as the Helping Alliance Questionnaire (*r* = 0.71) (Munder et al., 2009).

The *Real Relationship Inventory–Client–Short Form* (RRI–C–SF) (Stefana et al., 2024a) is an 8–item self–report measure of perception of the strength of the real relationship between patient and therapist from the perspective of the former. It contains two subscales of four items each: Realism and Genuineness. Ratings are made on a Likert scale, ranging from 1 (“Strongly disagree”) to 5 (“Strongly agree”). Higher scores indicate a stronger real relationship. In our study, Cronbach’s alpha and the average item correlation for the total scale were, respectively, 0.97 and 0.55. Real relationship has demonstrated strong convergence validity with both the construct of working alliance (*r* = 0.66 between RRI and total WAI scores) (Vaz et al., 2023) and the patient perceptions of genuineness or congruence (*r* = 0.71 with the dimension of congruence of the Barrett–Lennard Relationship Inventory) (Kelley et al., 2010).

The *in–Session Patient Affective Reactions Questionnaire* (SPARQ) (Stefana et al., 2023, 2024b) is an 8–item self–report questionnaire that assesses the perceptions and affective reactions of the patient toward his/her therapist during their individual psychotherapy session. It contains two subscales of four items each: Positive Affect (PA) and Negative Affect (NA). The SPARQ-PA scale captures the perception of a secure and comfortable relationship with his/her therapist from the perspective of the patient. The SPARQ-NA scale captures feelings of shyness, shame, fear of speaking openly, worry about not getting needed help, and a sense of failure due to his/her need for help from the therapist. Items are rated on a Likert scale ranging from 0 (“Not at all true”) to 4 (“Very true”). The two scales provide two distinct scores that cannot be summated with each other. In the current study, the Cronbach alpha values were 0.75 for the NA scale and 0.86 for the PA scale.

#### Session outcome domain

The *Session Evaluation Scale* (SES) (Hill and Kellems, 2002; Lent et al., 2006) is a 5–item self–report scale that assesses the perception of the quality of a therapy session. Four of the items are rated on a Likert scale ranging from 1 (“Strongly disagree”) to 5 (“Strongly agree”). These items require respondents to evaluate how pleased they were to have attended the most recent session, how satisfied they were with its outcome, and how helpful and valuable they found the session. An additional item is rated on a scale from 1 (“Very effective”) to 5 (“Ineffective”). The SES score is obtained by summing the values of the five items (after appropriate reversal is applied for two items) and then dividing by five. In our study, the SES showed an internal consistency Cronbach’s alpha value of 0.86.

### Statistical analyses

Categorical variables were reported as frequencies, while continuous variables were described using means, standard deviations, and ranges. Pearson’s correlation analyses were used to investigate relationships between the components of the TR, as measured by WAI-SR, RRI-C-SF, and SPARQ-PA and-NA, and session outcomes, as measured by the SES. Then a multiple regression analysis was performed to determine the unique contribution of each component of the TR in predicting the session outcome, with session outcome as the dependent variable and the four components of the TR as independent variables. To explore the interrelations between the four components of the TR, Pearson’s correlation coefficients were calculated. Furthermore, partial correlation analyzes were performed to control for possible confounder variables, which involved calculating correlation coefficients between the variables of interest, with the effect of treatment length partialed out. To examine the underlying structure of the components of the TR when their items were grouped together, exploratory factor analysis (EFA) was performed using a principal-axis factoring method and oblique rotation. The suitability of the data for factor analysis was evaluated using Bartlett’s sphericity test and the Kaiser-Meyer-Olkin test. Various methods, including the Hull method (Lorenzo-Seva et al., 2011), comparison data (Ruscio and Roche, 2012), Horn’s parallel analysis (Horn, 1965), and parallel analysis with principal component analysis and EFA, were employed to determine the optimal number of factors to retain. Items’ loadings were examined with a cutoff set at >0.30 (Raykov and Marcoulides, 2011). All analyzes were performed with R version 4.3.1.

### Procedure

Data was collected from September to November 2023 through two online patient registers: Research for Me and ResearchMatch. Research for Me has been developed by the North Carolina Translational and Clinical Sciences Institute, which represents the collaborative efforts of the US National Institutes of Health under the Clinical and Translational Science Awards (CTSA) Program at UNC-CH. Similarly, ResearchMatch (Harris et al., 2012) originates from a collaboration of leading academic institutions, receiving support from the US National Institutes of Health through the CTSA Program. To qualify, participants had to be adults 18 years or older and underwent individual psychotherapeutic treatment. They also needed to be fluent in English and capable of providing informed consent. After consenting, the participants underwent a baseline evaluation of their most recent therapy session. The survey was carried out using Qualtrics software.

## Results

Table 2 reports the mean, standard deviation, and item range for each measure of the TR used in this study. The SPARQ-NA exhibited positive skewness. Consequently, a logarithmic transformation was applied to the data and the natural logarithms of the values of the SPARQ-NA variable were used in all subsequent correlation and regression analyzes. The positively skewed distribution of the data for this variable is congruent with how we expect negative emotions toward the therapist to be experienced during sessions by the patients (i.e., rare to have strong negative feelings after most typical sessions). Moreover, despite the skewed distribution, the SPARQ-NA has demonstrated theoretically meaningful correlations with the other variables.

**Table 2:** Descriptive statistics and correlation matrix for study variables.

	1	2	2.1	2.2	2.3	3	4	5	5.1	5.2	Item range	Mean	SD
1. SES	1										1–5	4.06	0.84
2. WAI–SR	0.74	1									0–5	4.55	13.08
2.1 Goal	0.67	0.94	1								0–5	13.61	4.93
2.2 Task	0.72	0.92	0.84	1							0–5	12.46	4.80
2.3 Bond	0.63	0.87	0.72	0.68	1						0–5	14.47	4.61
3. SPARQ-PA	0.66	0.80	0.68	0.67	0.83	1					0–4	12.26	3.30
4. SPARQ-NA	−0.49	−0.49	−0.44	−0.46	−0.44	−0.49	1				0–4	3.18	3.13
5. RRI–C–SF	0.48	0.52	0.47	0.45	0.50	0.52	−0.38	1			1–5	31.49	6.65
5.1 Genuineness	0.44	0.44	0.40	0.40	0.40	0.44	−0.37	0.94	1		1–5	16.23	3.73
5.2 Realism	0.45	0.53	0.47	0.45	0.53	0.54	−0.34	0.93	0.75	1	1–5	15.26	3.37

RRI–C–SF, patient ratings of the real relationship inventory–short form; SES, session evaluation scale; SPARQ-NA, log transformations of patient ratings of the negative affect scale of the in–session patient affective reactions questionnaire; SPARQ-PA, patient ratings of the positive affect scale of the in–session patient affective reactions questionnaire; WAI–SR, patient ratings of the working alliance inventory–short revised. All the correlations are significant at *p* < 0.001.

Considering the varied settings of the psychotherapy sessions within the sample, items related to the setting of the session (either in–person face–to–face, in–person on the couch, by video call, or over the telephone) were examined for correlations with the components of the TR and the outcome of the session, as reported by the patients. This was to determine any potential associations between the setting of the session and the constructs of the study. The results did not reveal significant correlations between the setting of the session and the relational and outcome measures used in the study. Specifically, the correlation coefficients for the outcome measure (sestot_t0) ranged from −0.07 (telephone call) to 0.03 (in-person face-to-face), and for the relational measures, the correlation coefficients ranged from −0.08 (WAI bond in telephone call) to 0.08 (WAI task in telephone call).

A similar rationale was applied to investigate the impact of session frequency and duration of treatment on elements of TRs and session outcome. Analysis of variance (ANOVA) revealed that there were no significant differences in the means of TR or outcome measures across various session frequencies, namely, once a month or less, two to three times a month, once a week or two or more times a week. On the contrary, significant differences were observed in the means of TR and outcome measures over different durations of treatment. Specifically, WAI–SR, SPARQ-PA, and SES demonstrated significant differences at the 0.01 significance level between patients treated for 0 to 3 months and those treated for over 24 months. Furthermore, SPARQ-PA and SES exhibited significant differences at the 0.01 level between patients treated for 4–6 months and those treated for over 24 months. For all three measures, the trend is toward improvement with longer treatment durations. SPARQ-NA revealed significant differences between patients treated for 0 to 3 months (*p* < 0.01) or 4 to 6 months (*p* = 0.02) and those treated for more than 24 months. The trend for SPARQ Negative Affect is towards improvement (reduction in negative affect) with longer treatment durations, especially notable in patients treated for more than 24 months. RRI–C–SF did not show significant differences.

### Relationship between measures of therapeutic relationship and session outcome

The outcome of the patient-rated session outcome related positively to the patient-rated working alliance (*r* = 0.74, *p* < 0.001), real relationship (*r* = 0.48, *p* < 0.001), and positive affective reactions toward the therapist (*r* = 0.66, *p* < 0.001), and negatively to the negative affective reactions of the patient toward the therapist (*r* = −0.49, *p* < 0.001).

A simultaneous regression (Table 3) was conducted to examine the contributions of the working alliance, the real relationship and negative affective reactions toward the therapist, as perceived by the patients, to the patients’ ratings of session outcome. In this model, session outcome was the dependent variable. The results indicated that these four components together accounted for 30% of the variance in the session outcome (Adj.*R*^2^ = 0.58, *F* = 238, *p* < 0.001). All predictors in the model had a statistically significant effect on session outcome (see Table 2), with working alliance showing the strongest association (partial *r* = 0.53, *p* < 0.001) after adjusting for all other components. To explore the potential impact of the length of treatment, regression analyses were performed with treatment length partialed out. These analyzes showed that the relationships between variables remained significant and showed almost identical correlation values, indicating that the duration of treatment did not play a significant role in the relationships observed in this sample.

**Table 3:** Simultaneous regression model: therapeutic relationship components predicting session outcome.

	*B*	Std. Error	*β*	*t*	Significance level
Intercept	2.12	0.14		15.25	0.000
WAI–SR	0.03	0.00	0.53	12.47	0.000
SPARQ-PA	0.03	0.01	0.12	2.94	0.003
SPARQ-NA	−0.15	0.03	−0.14	−4.76	0.000
RRI–C–SF	0.01	0.00	0.09	2.95	0.003

RRI–C–SF, patient ratings of the real relationship inventory–short form; SES, session evaluation scale; SPARQ-NA, log transformations of patient ratings of the negative affect scale of the in–session patient affective reactions questionnaire; SPARQ-PA, patient ratings of the positive affect scale of the in–session patient affective reactions questionnaire; WAI–SR, patient ratings of the working alliance inventory–short revised.

### Interrelations between the four components of the therapeutic relationship

As hypothesized, there is a positive correlation between the working alliance and the real relationship (*r* = 0.48, *p* < 0.001) and the positive affective reactions towards the therapist (*r* = 0.80, *p* < 0.001), as well as between these latter two components (*r* = 0.52, *p* < 0.001). Furthermore, as hypothesized, the negative affective reactions of the patients towards the therapist were negatively related to both alliance (*r* = −0.49, *p* < 0.001), real relationship (*r* = −0.49, *p* < 0.001), and positive affective reactions (*r* = −0.49, *p* < 0.001).

To account for the significant variability in treatment length within our sample, we performed additional correlation analyses with treatment length partialed out. The results mirrored the original analysis in terms of magnitude and significance, indicating that the duration of treatment does not significantly influence the relationships among the components of the TR examined in this study.

### Factoring the model of the therapeutic relationship

The Bartlett test of sphericity (*p* < 0.001) and the Kaiser–Meyer–Olkin test (0.96) verified the suitability of the data for factor analysis. The Hull method (Lorenzo-Seva et al., 2011) and the parallel analysis with PCA and EFA all suggested retaining four factors. EFA revealed that the four–factor solution explained 64.3% of the variance. The four factors (considering items with factor loadings >0.30) closely resembled the original components: (i) working alliance, (ii) positive and (iii) negative affective reactions toward the therapist, and (iv) real relationship. The first factor emerging from the principal–axis factor analysis included the goal and task items of the working alliance, as well as the item (with a negative loading) “I felt worried my therapist could not help me,” which pertains to the negative affect reaction toward the therapist. The second factor perfectly corresponded to the real relationship. The third factor comprised all items of the positive affective reaction toward the therapist, in addition to the bond item of the working alliance. The fourth and final factor included the four items of the negative affect reaction toward the therapist. Table 4 (Model A) reports item loadings from exploratory factor analysis of combined TR components.

**Table 4:** Item loadings from exploratory factor analysis of combined therapeutic relationship components.

		Model A				Model B			
	Item content	F1	F2	F3	F4	F1	F2	F3	F4
Working alliance	As a result of this session, I am clearer as to how I might be able to change.	**0.80**	0.00	−0.03	0.01	**0.80**	−0.01	−0.06	−0.01
	What I am doing in therapy gives me new ways of looking at my problem.	**0.79**	0.03	0.05	0.06	**0.82**	0.03	−0.02	0.05
	I believe my therapist likes me.	0.09	0.05	**0.77**	−0.03	0.11	0.06	**0.76**	−0.06
	My therapist and I collaborate on setting goals for my therapy.	**0.73**	0.06	0.17	0.14	**0.76**	0.06	0.10	0.12
	My therapist and I respect each other.	0.20	0.05	**0.65**	−0.04	0.27	0.08	**0.54**	−0.07
	My therapist and I are working towards mutually agreed upon goals.	**0.76**	0.04	0.13	0.04	**0.78**	0.03	0.07	0.02
	I feel that my therapist appreciates me.	0.15	0.02	**0.81**	0.04	0.20	0.04	**0.75**	0.00
	My therapist and I agree on what is important for me to work on.	**0.67**	0.06	0.21	−0.01	**0.68**	0.06	0.17	−0.03
	I feel my therapist cares about me even when I do things that he/she does not approve of.	0.18	0.05	**0.66**	−0.07	0.20	0.06	**0.63**	−0.10
	I feel that the things I do in therapy will help me to accomplish the changes that I want.	**0.81**	0.07	0.05	−0.01	**0.84**	0.06	−0.02	−0.03
	My therapist and I have established a good understanding of the kind of changes that would be good for me.	**0.77**	0.08	0.11	0.03	**0.79**	0.07	0.05	0.02
	I believe the way we are working with my problem is correct.	**0.80**	0.07	0.06	−0.07	**0.83**	0.07	−0.01	−0.09
Positive affect	I felt happy to see my therapist.	0.28	0.10	**0.31**	−0.17	**0.33**	0.12	0.22	−0.19
	I felt my therapist cared about me.	0.08	0.07	**0.74**	−0.07	Item excluded			
	I felt respected by my therapist.	0.10	0.06	**0.65**	−0.13	Item excluded			
	I felt appreciated by my therapist.	0.06	0.08	**0.77**	−0.02	Item excluded			
Negative affect	I felt ashamed with my therapist about my fantasy, desires, mindset, behavior, or symptoms.	0.04	−0.05	−0.01	**0.54**	0.07	−0.04	−0.04	**0.56**
	I felt worried my therapist could not help me.	**−0.37**	−0.03	−0.07	**0.45**	−0.39	−0.03	−0.02	**0.47**
	I felt shy, like I wanted to hide from my therapist or end the session early.	0.02	−0.05	−0.08	**0.67**	0.03	−0.05	−0.06	**0.69**
	I felt afraid to spoke my mind, for fear of being judged, criticized, disliked by my therapist.	−0.06	−0.02	−0.06	**0.65**	−0.07	−0.03	−0.03	**0.66**
Real relationship	I was able to be myself with my therapist.	−0.04	**0.71**	−0.02	−0.13	−0.03	**0.71**	−0.10	−0.12
	I appreciated being able to express my feelings in therapy.	0.07	**0.84**	−0.07	−0.01	0.07	**0.85**	−0.14	0.00
	I was open and honest with my therapist.	0.02	**0.80**	−0.11	−0.19	0.01	**0.80**	−0.18	−0.18
	I was able to communicate my moment–to–moment inner experience to my therapist.	0.19	**0.64**	0.03	−0.07	0.20	**0.64**	−0.04	−0.07
	My therapist liked the real me.	−0.05	**0.76**	0.24	0.01	−0.06	**0.76**	0.20	0.01
	The relationship between my therapist and me was strengthened by our understanding of one another.	0.07	**0.65**	0.22	0.08	0.08	**0.65**	0.17	0.08
	I appreciated my therapist’s limitations and strengths.	0.08	**0.72**	0.14	0.11	0.07	**0.72**	0.11	0.12
	I had a realistic understanding of my therapist as a person.	0.02	**0.63**	0.19	0.13	0.03	**0.63**	0.14	0.14

The highest loadings for each factor are emphasized in bold.

Since three of the four bond items of the working alliance cover contents similar to three of the four items of positive affective reactions towards the therapist (correlation values were 0.79 for appreciation, 0.72 for respect, and 0.67 for care), we performed sensitivity analyses, conducted a parallel analysis and an exploratory factor analysis with an item pool that excluded the three items of the SPARQ. Analyses indicated four factors with the same pattern of loadings although the fourth factor had fewer items and was borderline in terms of size compared to parallel analysis (Table 4, Model B).

## Discussion

This study is among the few to have examined multiple elements of the TR simultaneously, from the patient’s perspective. The working alliance, the real relationship, and the positive and negative affective reactions toward the therapist together accounted for 30% of the variance in the outcomes of the session as rated by the patients. From the patient’s perspective, these four components of the TR were correlated with session outcomes to varying degrees. Moreover, they were found to be interrelated, yet distinctly separate entities. The findings of the present study add to the growing body of literature investigating the value of examining specific components of the TR.

We first investigated how the working alliance, the real relationship, and positive and negative affective reactions were correlated with the session outcome rated by the patients. Consistent with previous findings (Flückiger et al., 2020b), a stronger alliance was associated with a more positive session outcome. The magnitude of this correlation in our sample (*r* = 0.74) was consistent with that (*r* = 0.72) detected in a similar study that investigated the TR from the therapist’s perspective (Bhatia and Gelso, 2018). Not surprisingly, it was much stronger than the *r* = 0.23–31 found in meta-analyses of alliance–treatment outcome associations for adult psychotherapy (Flückiger et al., 2018, 2020a). This difference is explained by the fact that our outcome pertained to the session rather than the treatment outcome, as in the meta-analyses.

Similarly, patients who experienced positive affective reactions toward the therapist tended to perceive the session as of higher quality. The only previous study that has explored the correlation between session outcome (the same inventory used in the present study) and the positive affective response of the patient to the therapist as measured through the SPARQ-PA, showed a *r* of 0.64 (*n* = 475) (Stefana et al., 2024d). Furthermore, our finding of a large correlation value (*r* = 0.66) between the positive affective response and the session outcome is consistent with the correlation between the bond alliance dimension and the session outcome (*r* = 0.63). This similarity may be attributed to a significant overlap in the content of items measuring the bond dimension of the WAI-SR and the SPARQ-PA.

Patients who perceived a strong real relationship with their therapists were also likely to rate the session positively. This finding (*r* = 0.48) aligns with previous studies investigating this correlation from patients’ or therapists’ perspectives, which reported correlation coefficients ranging from 0.32 to 0.63 (Bhatia and Gelso, 2018; Marks et al., 2019; Pérez-Rojas and Gelso, 2020). These findings are consistent with existing meta-analytic findings (Gelso et al., 2018), which revealed an *r* of 0.38.

Lastly, the presence of negative reactions toward the therapist was associated with a lower quality of the session, as the patient rated. A previous study explored the association between SPARQ-NA and session outcomes, revealing similar results (*r* = −0.51) (Stefana et al., 2024d). Furthermore, the direction of our findings is theoretically meaningful and aligns with previous research on the correlation between therapist-rated session outcome and negative transference (Bhatia and Gelso, 2018). The difference in the magnitude of the correlations (*r* = −0.49 in our study versus *r* = 0.25 in previous research) may be explained by the likelihood that negative affective reactions encompass, but are not limited to, negative transference reactions.

In summary, the working alliance, the real relationship, and the positive and negative reactions of the patient toward the therapist are significantly related to the patient’s evaluation of the session outcome. A key finding of our study is that the negative affects experienced by patients toward their therapists during sessions are related to the session outcome with a lower magnitude than the positive affects. This suggests that in-session negative emotional reactions have a lesser impact on session quality compared to positive reactions. The working bond and the real relationship likely act as a buffer against negative affective reactions.

Although the simultaneous regression model primarily highlights the importance of the working alliance, the relevance of other components of the TR should not be prematurely dismissed. The roles of the real relationship, as well as positive and negative in-session reactions toward the therapist, may be better understood in relation to the working alliance. From the patient’s perspective, stronger in-session negative emotions are associated with weaker alliance and real relationship, aligning with the literature that examined negative transference (Marmarosh et al., 2009; Bhatia and Gelso, 2018). The interconnections between these components of the TR represent a potentially fruitful area of research.

In the present study, we made an initial attempt to understand the interconnections among the four elements of the TR by examining how items from the employed measures are grouped from the patient’s perspective. A principal-axis factor analysis revealed four factors that almost exactly corresponded to those posited by the respective measures. It is important to note that the SPARQ-PA and-NA tools use the same item format, and the formats of the other measures are also similar. Items of the bond dimension of the WAI-SR loaded on the same factor as items from the SPARQ-PA, but not on the factor with the RRI-C-SF items. This suggests that the bond of the alliance—an attachment reflecting the feelings and attitudes experienced by members of the therapeutic dyad toward each other (Hatcher and Barends, 2006; Pérez-Rojas et al., 2019)—differs substantially from the task and goal dimensions of the alliance, which are cognitive aspects emphasizing consensus or negotiation about therapy goals and the tasks required to achieve them (Mallinckrodt and Tekie, 2016).

The most significant theoretical (and potentially clinical) implication of this study’s findings pertains to the construct of alliance. Unlike the theory that alliance should be considered as comprising an emotional component (corresponding to the WAI bond dimension) and a cognitive component (corresponding to the WAI goals and tasks dimensions) (Wampold and Flückiger, 2023), we hypothesize that these components can and should be regarded as distinct constructs. This suggests that clinicians cannot simply focus on conveying information clearly and coherently. To increase the persuasive prominence of information, agreement on goals, and assignment of treatment tasks, it is crucial to invest in enhancing the bond dimension of the relationship (which is the patient’s emotional belief that their therapist cares for them, understands them, and will make efforts to support them). However, further research is needed to confirm this hypothesis.

Contrary to what has been theorized in the literature (Gelso, 2011), the working alliance and the real relationship inventory had no items that cross-loaded onto each other’s factors. More broadly, items from the real relationship did not show any non-trivial cross-loading, indicating that the bond dimension of factor 3 may be composed, at least to some extent, of transference and countertransference elements.

In general, factor analysis reveals how items from the four patient-rated TR measures used in this study coalesce into four distinct factors, demonstrating a limited yet theoretically meaningful degree of overlap. Findings offer support for a quadripartite model of integral elements from the patient’s perspective in the TR: the working alliance, real relationship, and positive and negative affective reactions towards the therapist. Theoretically, the positive and negative affective reactions of patients towards their therapist can be conceptualized as two specific patterns of transference (Westen and Gabbard, 2002; Bradley et al., 2005) that manifest within the TR. From a clinical perspective, these results highlight the importance for therapists of adopting a holistic approach that considers all components of the TR to optimize therapy outcomes.

### Limitations

The results of the present study should be considered in light of some limitations. First, the ecological validity of this study is limited by its cross-sectional design. The components of the TR can evolve throughout therapy (Gelso et al., 2012). Longitudinal research is necessary to elucidate how these components develop and interact over time. Second, our sample consisted only of self-selected participants who were informed about the general topic of the study: the TR. Consequently, their decision to participate may have been influenced by their personal views or experiences with therapy. Lastly, regarding future research, perspectives from therapists and/or external observers should be incorporated alongside those of the patients to provide a more comprehensive understanding.

## Conclusion

The findings of the present study sustain the importance of the relationship in healing. More specifically, they demonstrate that, from the patient’s perspective, the components of a quadripartite model of the TR encompassing the working alliance, real relationship, and positive and negative affective reactions towards the therapist account for a substantial amount of variance in session outcomes as rated by the patients. A deeper understanding of the relationship between the components of the TR and the outcomes of the session (and treatment) outcomes will enable the therapists to focus on those components that can improve or detract from the session outcome, both in terms of quality and effectiveness. Moreover, the results suggest that the affective bond dimension of the working alliance is a distinct construct from the cognitive task and goal dimensions. A crucial clinical takeaway is the importance of establishing and maintaining a positive alliance with patients.

## Data availability statement

The raw data supporting the conclusions of this article will be made available by the corresponding author upon reasonable request.

## Ethics statement

The Institutional Review Board of the University of North Carolina at Chapel Hill (UNC-CH) approved the study (IRB number: 23–0216; approval date: March 6, 2023). The study was conducted in accordance with the local legislation and institutional requirements. The participants provided their written informed consent to participate in this study.

## Author contributions

AS: Conceptualization, Formal analysis, Funding acquisition, Writing – original draft, Writing – review & editing. PF-P: Funding acquisition, Supervision, Writing – review & editing. EV: Supervision, Writing – review & editing. EY: Funding acquisition, Supervision, Writing – review & editing.
